# Effects of High-Pressure Homogenization on the Structure and Functional Properties of *Solenaia oleivora* Proteins

**DOI:** 10.3390/foods13182958

**Published:** 2024-09-18

**Authors:** Wanwen Chen, Xueyan Ma, Wu Jin, Haibo Wen, Gangchun Xu, Pao Xu, Hao Cheng

**Affiliations:** 1 Key Laboratory of Integrated Rice-Fish Farming Ecology, Ministry of Agriculture and Rural Affairs, Freshwater Fisheries Research Center, Chinese Academy of Fishery Sciences, Wuxi 214081, China; chenwanwen@ffrc.cn (W.C.); maxy@ffrc.cn (X.M.); jinw@ffrc.cn (W.J.); xugangchun@ffrc.cn (G.X.); xupao@ffrc.cn (P.X.); 2 Wuxi Fisheries College, Nanjing Agricultural University, Wuxi 214081, China; 3 Sino-US Cooperative International Laboratory for Germplasm Conservation and Utilization of Freshwater Mollusks, Freshwater Fisheries Research Center, Chinese Academy of Fishery Sciences, Wuxi 214081, China; 4 State Key Laboratory of Food Science and Resources, School of Food Science and Technology, Jiangnan University, Wuxi 214122, China

**Keywords:** *Solenaia oleivora* protein, high-pressure homogenization, structure, functional property, digestibility

## Abstract

*Solenaia oleivora*, a rare freshwater shellfish with high protein quality, is unique to China. However, the poor hydrosolubility and functional properties of *Solenaia oleivora* proteins hinder their utilization in food products. Herein, the alkaline dissolution-isoelectric precipitation method was used for the extraction of *Solenaia oleivora* proteins. Furthermore, the impact of high-pressure homogenization (HPH) treatment varying from 0 to 100 MPa on the structure and functional properties of *Solenaia oleivora* proteins was investigated. The obtained results indicated that HPH treatment decreased the α-helix content and enhanced the β-sheet and random coil content. Furthermore, the HPH caused the unfolding of protein structure, exposing aromatic amino acids, increasing the free thiol group content, and enhancing surface hydrophobicity. As the homogenization pressure increased from 0 to 100 MPa, the particle size of *Solenaia oleivora* proteins decreased from 899 to 197 nm with the polymer dispersity index (PDI) value decreased from 0.418 to 0.151, the ζ-potential increased from −22.82 to −43.26 mV, and the solubility increased from 9.54% to 89.96%. Owing to the significant changes in protein structure and solubility, the emulsifying, foaming, and digestive properties of *Solenaia oleivora* proteins have been significantly improved after treatment with HPH.

## 1. Introduction

The gradual growth in the global population with the rapid increase in the demand for protein-rich foods has stimulated the exploration of novel sustainable alternative protein sources [1]. Considering the limited land resources, aquatic creatures as an abundant source of high-quality proteins are becoming more precious [2]. Shellfish is one of the major aquatic resources, with an annual aquaculture output of 15.88 million tons in China in 2022. Besides the abundant resources, shellfish also possess the advantages of extraordinary nutrition, less greenhouse gas emissions, and a low risk of transmission of zoonotic diseases, as well as minimized food restriction induced by spiritual or religious reasons [3]. Thus, it is of great significance to utilize shellfish protein as an alternative source of renewable animal proteins. 

*Solenaia oleivora*, commonly referred to as the “abalone of the Huaihe River”, is classified under the class *Bivalvia* and belongs to the family *Unionidae*. It is a scarce and endangered freshwater edible shellfish endemic to China [4]. The previous studies mainly focused on transcriptome and metabolome analysis, clarifying the wound healing and immunity mechanisms of *Solenaia oleivora* related to the breeding areas [4,5,6]. It is worth noting that the Chinese Academy of Fishery Sciences’ Freshwater Fisheries Research Centre has overcome the artificial breeding technology of *Solenaia oleivora* through in vitro culture since 2020 [7]. *Solenaia oleivora* possesses higher protein content (up to 12% of wet weight in edible portions) than *Hyriopsis cumingii*, *Sinonovacula constricta*, *Meretrix meretrix Linnaeus*, *Solen graudis*, oysters, and other shellfish [8]. However, little research has been carried out on the extraction and modification of *Solenaia oleivora* protein. Like the other invertebrate muscles, the basic proteins of shellfish mainly consist of actin, myosin, and collagen [9]. The functional properties of shellfish proteins mainly depend on their structures, while the poor solubility of shellfish proteins at low ionic strength due to the complex structure results in lower functional properties, further restricting their applications as human food. 

High-pressure homogenization (HPH) has attracted much attention in creating desirable functional properties of food proteins due to the advantages of low cost and high efficiency. The pressure, number of cycles, flow rate, and temperature are the key parameters affecting the protein structures of fluid samples during the HPH process [10]. The combination of physical effects exerted by HPH, such as intense shear force, high-frequency turbulence, convective collision, and cavitation forces, induce the alteration in protein structures, further improving their performance (e.g., solubility, emulsifying capacity, and foaming ability) as food ingredients [11]. The mechanical forces of HPH can disrupt intramolecular and intermolecular interactions of proteins, leading to conformational changes in the secondary, tertiary, and quaternary structures of proteins. It can cause the dissociation of insoluble protein aggregates, resulting in the exposure of hydrophobic and hydrophilic moieties, further enhancing the physicochemical and functional properties of protein aggregates [12]. A few studies have reported that HPH treatment can modify the structure and enhance the functional properties of several shellfish proteins, especially in oysters (*Crassostrea gigas*) [13,14], mussels (*Mytilus edulis*) [2,15], clams (*Ruditapes philippinarum*) [16], and scallops (*Chlamys farreri*) [17]. Notably, shellfish proteins from various species have different sensitivities to homogeneous pressure and cycle number. Meanwhile, high-intensity and continuous high-pressure homogenization will exert side effects, including thermal denaturation, severe aggregation, and a decrease in solubility and functional properties. For example, the solubility of oyster protein significantly increased from 31.07% to 84.7% when the homogenization pressure increased from 0.1 to 100 MPa, while maximal emulsifying and foaming properties occurred after treatment at 20 MPa [18]. As for the proteins from clams (*Ruditapes philippinarum*), the solubility reached its maximum value of 70.8% at 80 MPa homogenization pressure, and a decrease was observed when the homogenization pressure was continually increased to 100 MPa. The homogenization pressure that ranged from 40 to 100 MPa showed no appreciable influence on the foaming ability of the proteins from clams (*Ruditapes philippinarum*) [16]. Thus, it is of great importance to explore the optimal conditions of HPH treatment for the structural modification and functional property improvement of shellfish proteins. 

In the present study, the influence of HPH on the structure, functional characteristics, and digestive properties of *Solenaia oleivora* protein was investigated. Additionally, the relationship between the structural features and functional attributes of *Solenaia oleivora* protein was elucidated. This study not only provides a better understanding of the modifications in physicochemical and functional characteristics of *Solenaia oleivora* protein during HPH processing but also gives a theoretical foundation for the exploitation of *Solenaia oleivora* protein as a new food protein resource.

## 2. Materials and Methods

### 2.1. Materials 

Fresh *Solenaia oleivora* were obtained from Funan Jinghuai Special Aquatic Products Co., Ltd. (Funan, China). Pancreatin (4× USP specifications), pepsin (≥500 U/mg), 5, 5′-dithiobis (2-nitrobenzoic acid) (DTNB), and 8-anilino-1-naphthalenesulfonic acid (ANS) were obtained from Sigma-Aldrich Co., Ltd. (St. Louis, MO, USA). Other chemicals were of analytical purity and obtained from SinoPharm Chemical Reagent Co., Ltd. (Shanghai, China).

### 2.2. Protein Extraction

The *Solenaia oleivora* protein was prepared according to the method described by Chang et al. [19]. Previously reported studies suggested that the water-to-meat ratio varied from 3:1 to 9:1 and precipitation pH varied from 4.5 to 5.5 were recommended for the mussel protein extraction [15,20,21]. In this study, the edible part of the *Solenaia oleivora* was washed, crushed, and dispersed in pre-cooled deionized water at a *Solenaia oleivora*/water ratio of 1:4, 1:6, and 1:8 g/mL. Then, the dispersion was homogenized for 2 min, and the pH was adjusted to 12.0. The mixture was stirred for 60 min and subsequently subjected to centrifugation at 10,000× *g* for 15 min. Moreover, 2 M HCl was used to adjust the pH of the supernatant to 4.5, 5.0, and 5.5, and the dispersion was centrifuged at 10,000× *g* for 15 min. The precipitation was redispersed in deionized water, and the pH of the solution was neutralized to 7.0 and then freeze-dried. The protein content of the obtained freeze-dried powder of *Solenaia oleivora* protein was determined by the Kjeldahl nitrogen approach with a protein conversion coefficient of 6.25. The yield of protein was determined by the following formula:

(1) $$\mathrm{Protein}\mathrm{yield}\;\left(\%\right)=\frac{\mathrm{The}\;\mathrm{mass}\;\mathrm{of}\mathrm{protein}\;\mathrm{in}\;\mathrm{freeze-dried}\;\mathrm{powder}}{\mathrm{The}\;\mathrm{mass}\;\mathrm{of}\;\mathrm{Solenaia}\;\mathrm{oleivora}\;\mathrm{meat}}$$

### 2.3. High-Pressure Homogenization Treatment

A total of 10 mg/mL *Solenaia oleivora* protein dispersion was treated under the homogeneous pressure varied from 0 to 100 MPa for 3 min using an AH-2100 high-pressure homogenizer (ATS Engineering Co., Ltd., Brampton, ON, Canada) [13]. Samples after homogenization treatment were stored at 4 °C or lyophilized by a Freezone freeze dryer (LABCONCO Co., Ltd., Kansas, MO, USA).

### 2.4. Structural Measurement

#### 2.4.1. Circular Dichroism (CD)

The CD spectra of the proteins were determined using a JASCO-1700 circular dichroism spectrometer (JASCO Corporation, Tokyo, Japan) based on the approach reported by Cheng et al. [22]. The CD spectra of *Solenaia oleivora* protein solutions at the concentration of 0.25 mg/mL were measured at the range of 190–260 nm with a bandwidth of 1.0 nm and scanning speed of 50 nm/min. Secondary structure content was determined according to Yang’s method [2].

#### 2.4.2. Fluorescence Spectra 

The fluorescence spectra were analyzed on a fluorescence spectrophotometer (Agilent Co., Ltd., New York, NY, USA). A total of 0.20 mg/mL *Solenaia oleivora* protein solution was excited at 280 nm, and the emission spectra were captured from 290 to 550 nm with the excitation and emission slits set at 5 nm. 

#### 2.4.3. Free Sulfhydryl Group 

The free sulfhydryl group content of the *Solenaia oleivora* proteins was determined using the previously reported Ellman’s method with slight modifications [23]. A total of 2 mg/mL *Solenaia oleivora* protein solution was dispersed in a phosphate buffer solution containing 1 mM EDTA or 1 mM EDTA and 6 M urea, respectively. Then 50 μL of 4 mg/mL DTNB solution was added, and the vortexed mixture was kept at 25 °C for 60 min in darkness. The absorbance of the sample at 412 nm was determined using a UV-1800 ultraviolet spectrophotometer (Shimadzu Corporation, Kyoto, Japan). The free sulfhydryl group content was calculated by the following formula.

(2) $$\mathrm{Sulfhydryl}\;\mathrm{group}\;\mathrm{content}\;(\mu \mathrm{mol}/g)=\frac{A_{412}\;\times \;D\;\times \;10^{6}}{C\;\times \;13,600}$$

where A_412_ is the absorbance value at 412 nm, D is the dilution factor, C is the protein concentration (mg/mL), 13,600 is a molar extinction coefficient (M^−1^ cm^−1^). 

#### 2.4.4. Surface Hydrophobicity (H_0_)

The H_0_ was analyzed on a fluorescence spectrophotometer (Agilent Technologies, New York, NY, USA) using ANS as a fluorescence probe according to the previously reported method [24]. Briefly, 50 μL of 8 mM ANS solution at pH 7.0 was added to *Solenaia oleivora* protein solution at the concentration of 0.01–0.10 mg/mL. Fluorescence intensity was measured at an emission wavelength of 470 nm with an excitation wavelength set at 390 nm. H_0_ was expressed as the initial slope of fluorescence intensity versus *Solenaia oleivora* protein concentration (mg/mL). 

#### 2.4.5. Particle Size and ζ-Potential

The particle size, PDI, and ζ-potential of the *Solenaia oleivora* protein solution were detected by a NanoBrook Omni particle size analyzer (Brookhaven Instruments Corporation, Holtsville, NY, USA) at 25 °C. The detection parameters were set as follows: the equilibration time was 60 s, the detection angle was 90°, the refractive index of water was 1.331, and the refractive index of protein was 1.450. The ζ-potential was calculated using the Smoluchowski model.

### 2.5. Solubility and Turbidity Measurement

#### 2.5.1. Solubility Measurement

The *Solenaia oleivora* protein was diluted to the concentration of 5 mg/mL and centrifuged at 10,000× *g* for 15 min. The Kjeldahl nitrogen method was applied for the determination of protein content before and after centrifugation. The protein solubility (%) was calculated by Formula (3):

(3) $$S\mathrm{olubility}\left(100\%\right)=\frac{\mathrm{The}\;\mathrm{content}\;\mathrm{of}\;\mathrm{nitrogen}\;\mathrm{in}\;\mathrm{supernatant}}{\mathrm{The}\;\mathrm{content}\;\mathrm{of}\;\mathrm{nitrogen}\;\mathrm{in}\;\mathrm{the}\;\mathrm{sample}}\times 100\%$$

#### 2.5.2. Determination of Turbidity

The concentration of the *Solenaia oleivora* protein sample was diluted to 5 mg/mL. The transmittance (T%) of different samples at 600 nm was determined using a UV-1800 ultraviolet-visible spectrophotometer. The turbidity of the sample was expressed as 100%-T%.

### 2.6. Functional Properties Analysis 

#### 2.6.1. Emulsifying Properties

The emulsifying activity and stability indices of *Solenaia oleivora* protein were assessed based on the method as described by Malik et al. [25]. A total of 5 mL sunflower oil was added in 15 mL 2 mg/mL of the *Solenaia oleivora* protein and homogenized at 10,000 rpm for 2 min. The emulsion was centrifuged at 1500× *g* for 5 min. To evaluate the stability of the emulsion, the emulsion was heated to 80 °C for 30 min before centrifugation. The emulsifying properties were calculated by the following formula:

(4) $$\mathrm{Emulsifying}\;\mathrm{activity}\;\left(\%\right)=\frac{\mathrm{Height}\;\mathrm{of}\;\mathrm{the}\;\mathrm{emulsified}\;\mathrm{layer}}{\mathrm{Height}\;\mathrm{of}\;\mathrm{total}\;\mathrm{content}\;\mathrm{in}\;\mathrm{the}\;\mathrm{tube}}\;\times 100$$

(5) $$\mathrm{Emulsifying}\;\mathrm{stability}\;\left(\%\right)=\frac{\mathrm{Height}\;\mathrm{of}\;\mathrm{emulsified}\;\mathrm{layer}\;\mathrm{after}\;\mathrm{heating}}{\mathrm{Height}\;\mathrm{of}\;\mathrm{total}\;\mathrm{content}\;\mathrm{in}\;\mathrm{the}\;\mathrm{tube}\;\mathrm{after}\;\mathrm{heating}}\;\times 100$$

#### 2.6.2. Foaming Properties 

The foaming properties of *Solenaia oleivora* protein were analyzed refer to the previously reported approach [2]. A total of 2 mg/mL *Solenaia oleivora* protein solution was stirred vigorously for 2 min. The volume of foam was measured immediately. The changes in the volume of foam were also detected after standing for 20 min at room temperature. The measurement of foaming capacity and foam stability was conducted using the following formula:

(6) $$\mathrm{Foaming}\;\mathrm{capacity}\;\left(\%\right)=\frac{\mathrm{Volume}\;\mathrm{after}\;\mathrm{whipping-volume}\;\mathrm{before}\;\mathrm{whipping}}{\mathrm{Volume}\;\mathrm{before}\;\mathrm{whipping}}\times 100\;$$

(7) $$\mathrm{Foam}\;\mathrm{stability}\;\left(\%\right)=\frac{\mathrm{Volume}\;\mathrm{after}\;\mathrm{standing}\;\mathrm{time}}{\mathrm{Initial}\;\mathrm{volume}}\times 100\;$$

#### 2.6.3. Determination of In Vitro Digestibility 

The INFOGEST in vitro simulated gastrointestinal digestion model was used to evaluate the digestibility of *Solenaia oleivora* protein as the methods described by Garvey et al. [26]. A total of 5 mg/mL of the *Solenaia oleivora* protein solution was first incubated with simulated gastric fluid (SGF, pH 3.0, 2000 U/mL pepsin) at a volume ratio of 1:1 for 2 h and then mixed with simulated intestinal fluid (SIF, pH 7.0, 100 U/mL pancreatin) at a volume ratio of 1:4 for 2 h. The digestive fluids at digestive times of 15, 30, 60, 120, 180, and 240 min were collected, and the enzyme deactivation was carried out in a boiling water bath. Then, 30% trichloroacetic acid was added to the mixture, and the protein precipitate was obtained by centrifugation at 10,000× *g* for 15 min. A total of 1 mL of 1 M NaOH was used to redissolve the protein, and the protein content was measured using the Kjeldahl nitrogen method. The protein digestibility was calculated using Formula (8):

(8) $$\mathrm{Protein}\;\mathrm{digestibility}\;\left(\%\right)=\frac{C_{t}\;-\;C_{d}}{C_{t}}\;\times 100$$

where C_t_ represents the protein concentration prior to digestion, and C_d_ denotes the protein concentration following digestion.

### 2.7. Statistical Analysis

Each experiment was repeated three times, with the findings reported as the average value ± standard deviation. One-way analysis of variance (ANOVA) with the Duncan model was performed to conduct the statistical analysis using SPSS 20.0 software. *p* < 0.05 indicated a significant difference.

## 3. Results and Discussion

### 3.1. Extraction of Solenaia oleivora Protein

The extraction efficiency of shellfish protein by the alkali dissolution-isoelectric precipitation method is mainly related to factors such as the liquid/solid ratio, pH of alkali dissolution, and pH of precipitation. [27]. Previous studies have shown that when the alkali dissolution pH is 12.0, the content of the extracted shellfish protein is the highest [15]. Therefore, this study investigated the effects of liquid/solid ratio and the pH of precipitation on the extraction rate and purity. It was found that under the same pH of precipitation condition, the extraction rate gradually increased as the liquid/solid ratio increased from 4:1 to 8:1, while the purity of the obtained protein slightly decreased (Table 1). At the same liquid/solid ratio, the extraction rate and purity of protein significantly improved as the pH of precipitation increased from 4.5 to 5.5. Among them, the extracted *Solenaia oleivora* protein had the highest purity of 95.44% at the liquid/solid ratio of 6:1. As the liquid/solid ratio increased to 8:1, a maximum value of 93.92% occurred in the protein yield.

### 3.2. Effects of Homogeneous Pressure on the Structural of Solenaia oleivora Protein

#### 3.2.1. Secondary Structure Analysis

α-Helix structure in *Solenaia oleivora* protein decreased from 31.3% to 19.1% as the homogeneous pressure increased up to 100 MPa (Table 2). At the same time, β-turn structure increased from 13.5% to 21.2%, and random coil content increased from 39.5% to 43.1%. However, HPH treatment did not significantly influence β-sheet structure content in *Solenaia oleivora* protein. The results indicated that HPH treatment could significantly change the secondary structure of *Solenaia oleivora* protein, which was similar to the trend of secondary structure changes in oyster protein [28]. This may be due to the unfolding of the protein structure induced by HPH treatment, leading to the disruption of hydrogen bonds, further resulting in the transformation of the α-helix to random coils and β-turn structures [29].

#### 3.2.2. Fluorescence Spectra Analysis

**Figure 1:**
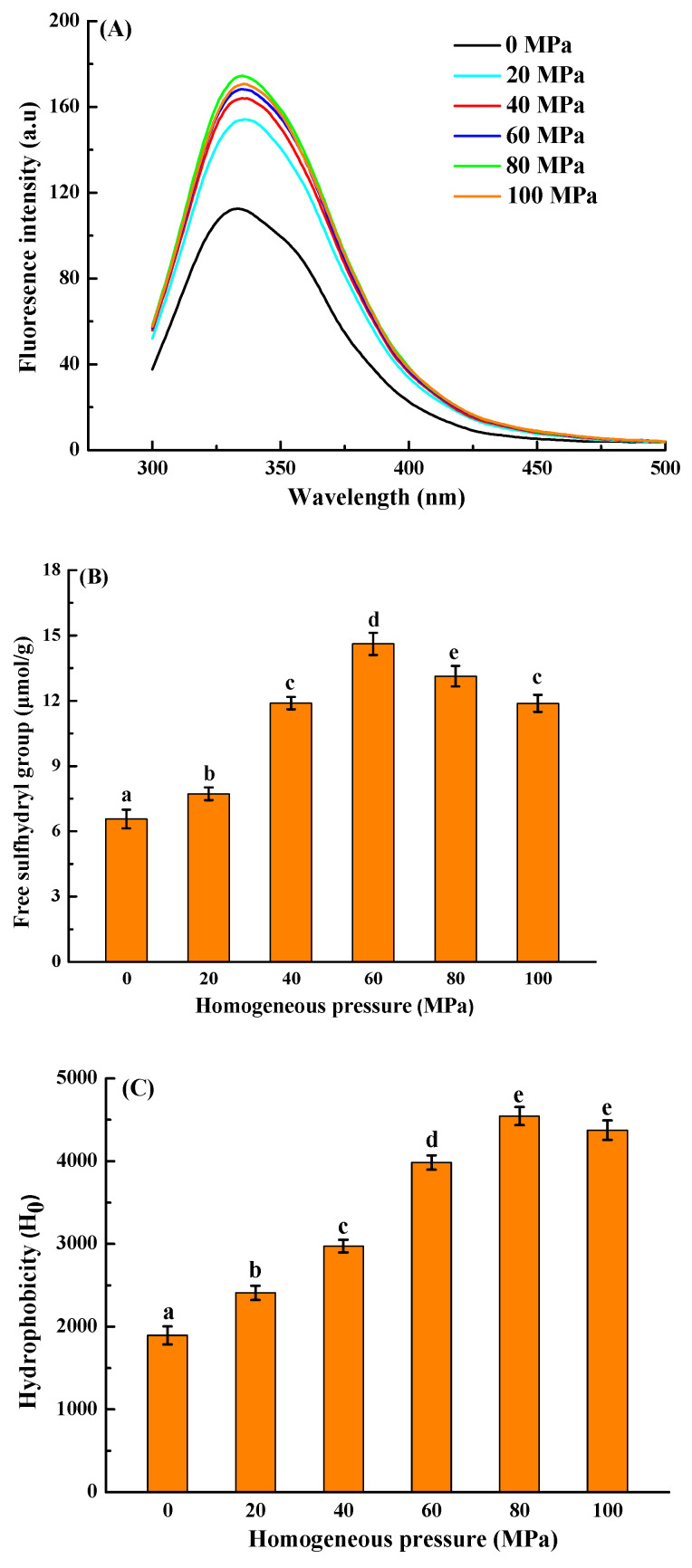
Impact of homogenization pressure on the fluorescence spectra (**A**), free sulfhydryl group content (**B**), and surface hydrophobicity (**C**) of *Solenaia oleivora* proteins. Different letters represent significant differences at *p* < 0.05.

Intrinsic fluorescence spectroscopy is an effective approach for detecting the positions of chromophores, reflecting the tertiary structure changes [30]. HPH treatment caused a red shift in the maximum emissions wavelengths (λ_max_) from 333 to 337 nm as the homogeneous pressure increased from 0 to 80 MPa (Figure 1A). Moreover, a 1.54-fold increase in intensity was also observed under the same conditions. The results revealed that the HPH treatment caused the partial structure unfolding and induced the transfer of more tryptophan residues to the hydrophilic environment [31]. It suggested that the tertiary structures of *Solenaia oleivora* protein were susceptible to HPH treatment. 

#### 3.2.3. Sulfhydryl Content Analysis

Figure 1B shows the change in free sulfhydryl content in *Solenaia oleivora* protein during HPH treatment. When the homogeneous pressure increased up to 60 MPa, the free sulfhydryl group content in the *Solenaia oleivora* protein significantly increased from 6.56 to 14.61 μmol/g, which was mainly because HPH treatment induced the development of protein structure and led to the exposure of sulfhydryl group in protein molecules [2]. When the homogenizing pressure continuously increased to 100 MPa, the free sulfhydryl group content gradually decreased to 11.88 μmol/g. In the HPH process, the increased free sulfhydryl group content can oxidize to form a disulfide bond under aerobic conditions, resulting in a decrease in free sulfhydryl group content in *Solenaia oleivora* protein [15,32].

#### 3.2.4. Surface Hydrophobicity Analysis

Surface hydrophobicity (H_0_) can reflect the relative content of hydrophobic groups on the surface of protein or aggregates in contact with a hydrophilic environment. When the homogenization pressure increased up to 80 MPa, the surface hydrophobicity of the protein gradually increased from 1894 to 4545 (Figure 1C), indicating that the molecular structure of the protein was unfolded during the homogenization process, and the hydrophobic groups buried in the protein were gradually exposed. It was consistent with the fluorescence spectrum results in Figure 1A. Continuing to increase the homogeneous pressure to 100 MPa slightly reduced the hydrophobicity of the *Solenaia oleivora* protein, which may be due to the reassembly of the protein molecules at higher homogeneous pressure and the re-formation of protein aggregates, resulting in a decrease in the exposed hydrophobic groups.

#### 3.2.5. Particle Size and ζ-Potential Analysis

**Figure 2:**
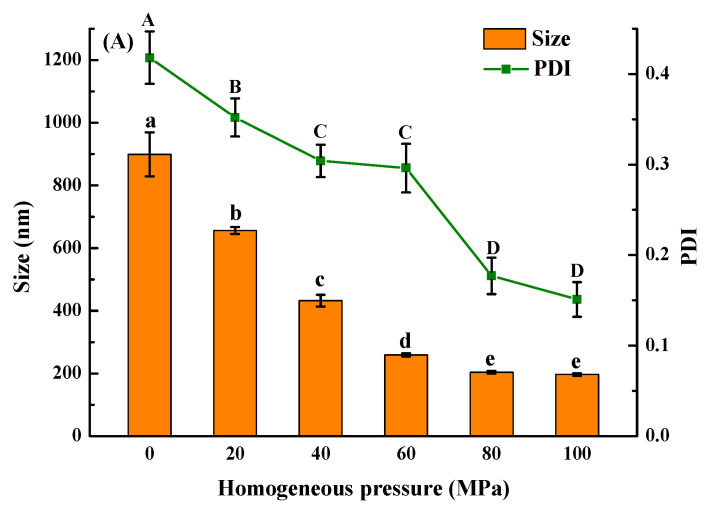

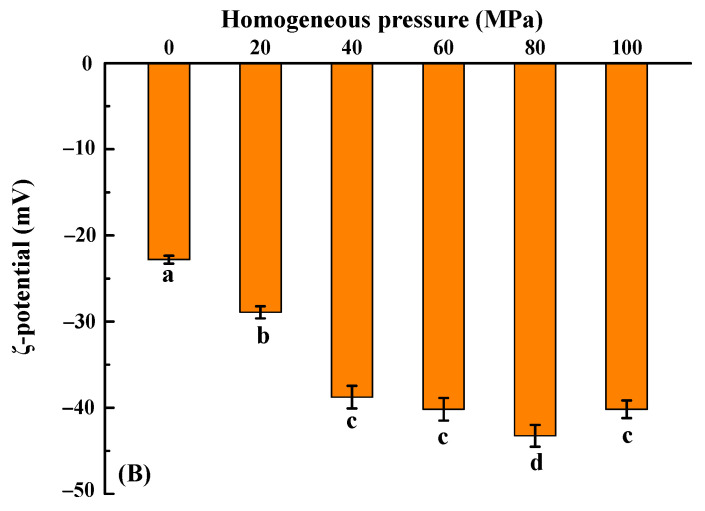
Effect of homogenization pressure on the mean particle size and PDI (**A**) and ζ-potential (**B**) of *Solenaia oleivora* proteins. Different letters represent significant differences at *p* < 0.05.

The protein solution of *Solenaia oleivora* without HPH treatment had a large average particle size of 899 nm with a PDI value of 0.418, respectively (Figure 2A). The average particle size and PDI value of protein particles decreased as the homogeneous pressure increased. When the homogeneous pressure reached 80 MPa, the mean particle size and PDI were 204 nm and 0.177, respectively, indicating a relatively uniform particle size distribution. However, no significant changes were observed as the homogeneous pressure continuously increased to 100 MPa. The decrease in the protein particle size is mainly due to the cavitation effect induced by HPH and the high hydrodynamic shear force destroying the non-covalent interactions between protein molecules [17].

The *Solenaia oleivora* protein without HPH treatment had a negative charge of 22.82 mV (Figure 2B). The absolute value of ζ-potential increased with the increasing homogeneous pressure. When the homogeneous pressure was 80 MPa, the ζ-potential reached a maximum value of −43.26 mV, which may be ascribed to the revelation of charged amino acid residues. Owing to the steric hindrance effect, it is generally believed that the absolute value of ζ-potential higher than 20 mV could offer sufficient electrostatic repulsion for maintaining the physical stability of protein aggregates [33].

### 3.3. Solubility and Turbidity Analysis

**Figure 3:**
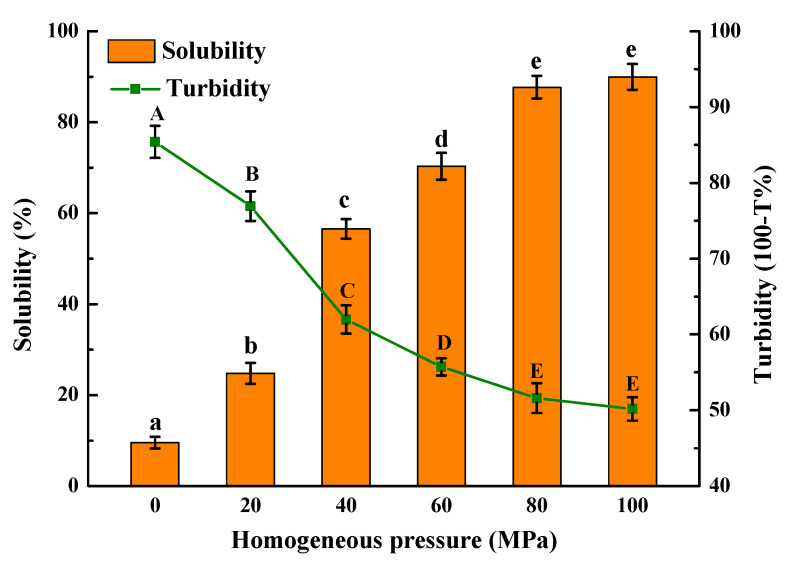
Effect of homogenization pressure on the solubility and turbidity of *Solenaia oleivora* proteins. Different letters represent significant differences at *p* < 0.05.

It was found that the *Solenaia oleivora* protein had a lower solubility of 9.52% and larger turbidity of 85.42%, respectively (Figure 3). With the increase in homogeneous pressure to 100 MPa, the protein solubility increased significantly with a maximum value of 89.96%, and the protein turbidity decreased significantly with a minimum value of 50.18%. The above results indicated that HPH could significantly improve the solubility of *Solenaia oleivora* protein. Some studies have shown that the surface hydrophobicity of proteins will be increased under high homogeneous pressure, leading to insoluble protein aggregate formation and decreased hydrosolubility [34]. However, in this study, such an “over-processing” effect was not detected under the high homogeneous pressure of 100 MPa. Protein solubility is dependent on protein structure and conformation, particle size, exposed functional groups, and surface hydrophobicity [13]. In this study, the ζ-potential value of *Solenaia oleivora* protein varied from −39 to −43 mV as the homogenization pressure increased from 40 to 100 MPa (Figure 2B), probably giving rise to a negligible effect on the protein solubility. Meanwhile, the increased homogenization pressure caused the continuous reduction in the particle size and PDI of protein particles (Figure 2A), promoting the ability of protein–water interaction and the formation of soluble and small protein aggregates with better dispersion or hydration [14]. Therefore, the enhancement in protein solubility due to HPH treatment is primarily attributed to the reduction in protein aggregate particle size caused by the cavitation effect and high-speed shear force during the HPH process [2]. At the same time, the unfolding of the protein exposes more hydrophilic groups, increasing the interaction between water and protein and leading to the improvement of solubility [35].

### 3.4. Emulsification and Foaming Characteristics

**Figure 4:**
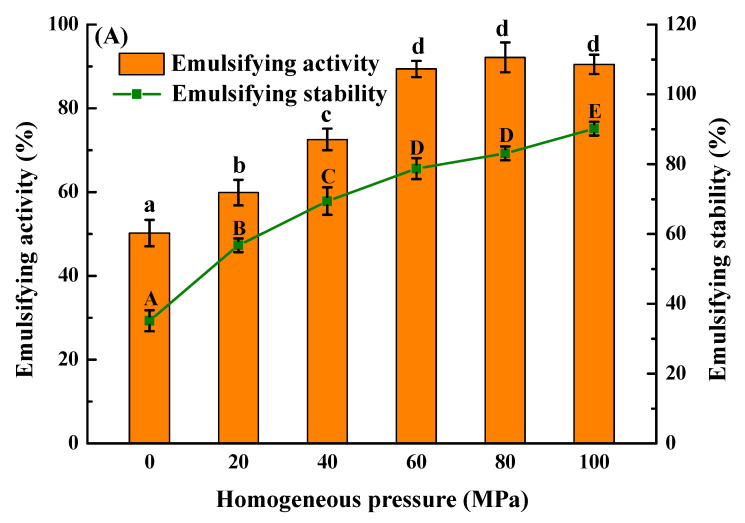

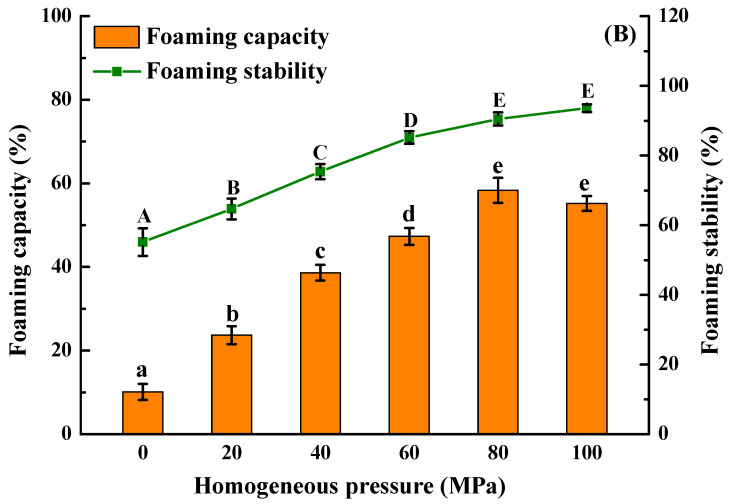
Effect of homogenization pressure on emulsifying properties (**A**) and foaming properties (**B**) of *Solenaia oleivora* proteins. Different letters represent significant differences at *p* < 0.05.

The emulsification properties and emulsification stability of *Solenaia oleivora* protein were increased by about 42% and 55% after being treated with a homogeneous pressure of 100 MPa (Figure 4). The foaming ability and foam stability of *Solenaia oleivora* protein also increased by 45% and 38% at the same homogeneous pressure as compared to the control group. This indicated that HPH treatment can significantly improve the emulsifying and foaming properties. According to the results obtained in the solubility and surface hydrophobicity, HPH treatment can significantly increase the solubility of *Solenaia oleivora* protein and reduce its particle size, which is conducive to the rapid diffusion and adsorption of protein aggregates to water–oil and water–gas interfaces, thus increasing the emulsification and foaming ability [36]. At the same time, the elevated surface charge and the interfacial viscoelastic film formation may aid in enhancing the stability of emulsified droplets and bubbles.

### 3.5. In Vitro Digestibility Analysis

**Figure 5:**
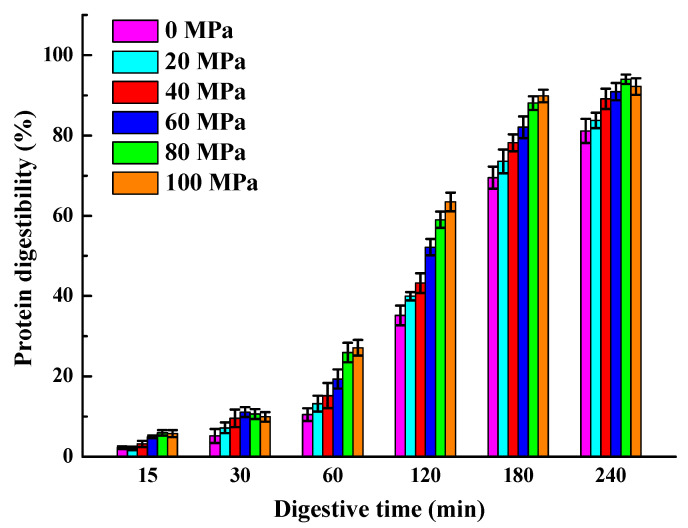
Effect of homogenization pressure on the in vitro digestive properties of *Solenaia oleivora* proteins.

As shown in Figure 5, the in vitro digestibility of natural *Solenaia oleivora* protein without HPH treatment was 82%, indicating that *Solenaia oleivora* protein had good in vitro digestion characteristics. HPH treatment increased the rate and degree of protein digestion. The improvement effect is more obvious with the gradual increase in homogeneous pressure, and the digestibility can reach 94%. During HPH treatment, more aromatic amino acid residues are produced, while pepsin tends to hydrolyze aromatic amino acid residues [37], thus improving the in vitro digestibility of *Solenaia oleivora* proteins. In addition, HPH treatment also caused a decrease in particle size and an increase in solubility of the protein aggregates of *Solenaia oleivora*, resulting in a relatively high specific surface area and diffusion rate, which further improved the hydrolysis efficiency of digestive enzymes.

## 4. Conclusions

The exploration of renewable proteins and the expansion of their utilization have attracted great attention. In our study, the *Solenaia oleivora* protein was prepared by alkaline-isoelectric precipitation methods. The effects of HPH on the structure, function, and digestion of the protein were investigated. The results showed that high-pressure homogenization can significantly change the structure of the *Solenaia oleivora* protein, resulting in the expansion of the protein structure, the increase in the free sulfhydryl group, and surface hydrophobicity. Meanwhile, HPH can significantly decrease the particle size and increase the ζ-potential of the protein aggregates. These changes in structure and size significantly improved the solubility, emulsification, foaming, and in vitro digestion characteristics of the *Solenaia oleivora* protein. This study provides a new resource of proteins from freshwater shellfish and suggests a strategy to expand the utilization of *Solenaia oleivora* protein in the food industry. 

## Figures and Tables

**Table 1:** Purity and yield of *Solenaia oleivora* proteins under different liquid/solid ratios and precipitated pH values.

Liquid/Solid Ratio	pH	Protein Purity (%)	Protein Yield (%)
4:1	4.5	83.44 ± 0.93 ^bc^	72.58 ± 1.49 ^a^
	5.0	85.44 ± 1.83 ^cd^	76.61 ± 1.27 ^b^
	5.5	92.44 ± 2.24 ^e^	81.24 ± 1.71 ^c^
6:1	4.5	82.13 ± 0.92 ^b^	75.29 ± 1.36 ^b^
	5.0	86.86 ± 1.11 ^d^	80.32 ± 1.56 ^c^
	5.5	95.44 ± 1.26 ^f^	88.92 ± 1.43 ^e^
8:1	4.5	79.39 ± 2.14 ^a^	79.13 ± 0.92 ^c^
	5.0	83.19 ± 2.08 ^bc^	84.87 ± 0.73 ^d^
	5.5	87.58 ± 0.87 ^d^	93.92 ± 0.98 ^f^

Data were presented as Mean ± SD, n = 3. Different letters within a column represent significant differences at *p* < 0.05.

**Table 2:** Effect of homogenization pressure on the secondary structure content of *Solenaia oleivora* proteins.

Homogeneous Pressure (MPa)	α-Helix (%)	β-Sheet (%)	β-Tum (%)	Random Coil (%)
0	31.3 ± 2.6 ^a^	15.7 ± 1.4 ^a^	13.5 ± 1.6 ^a^	39.5 ± 0.9 ^a^
20	28.7 ± 1.6 ^ab^	16.8 ± 0.8 ^a^	14.4 ± 0.6 ^a^	40.1 ± 1.5 ^a^
40	26.1 ± 1.3 ^b^	15.9 ± 1.8 ^a^	17.1 ± 0.5 ^b^	40.9 ± 1.0 ^a^
60	22.1 ± 1.5 ^c^	16.7 ± 1.0 ^a^	18.6 ± 0.6 ^bc^	42.6 ± 0.2 ^b^
80	20.9 ± 1.2 ^c^	16.5 ± 0.7 ^a^	19.7 ± 0.6 ^cd^	42.9 ± 0.8 ^b^
100	19.1 ± 1.1 ^c^	16.6 ± 1.0 ^a^	21.2 ± 1.4 ^d^	43.1 ± 0.8 ^b^

Data were presented as Mean ± SD, n = 3. Different letters within a column represent significant differences at *p* < 0.05.

## Data Availability

The data presented in this study are available on request from the corresponding author.
